# Nanoarchitectonics of Injectable Biomimetic Conjugates for Cartilage Protection and Therapy Based on Degenerative Osteoarthritis Progression

**DOI:** 10.34133/bmr.0075

**Published:** 2024-09-10

**Authors:** Jingwei Bi, Limin Zhang, Pengfei Zhang, Shulei Xu, Yuhao Liu, Xiaolai Zhang, Xiaoyong Qiu, Yanwen Bi, Fangfang Yan, Hui Wei, Xin Cui, Xin Pan, Jun Huang, Yunpeng Zhao

**Affiliations:** ^1^ Department of Orthopaedic, Qilu Hospital of Shandong University, Jinan, Shandong 250012, China.; ^2^ Center for Advanced Jet Engineering Technologies (CaJET), Key Laboratory of High Efficiency and Clean Mechanical Manufacture of Ministry of Education, School of Mechanical Engineering, Shandong University, Jinan, Shandong 250061, China.; ^3^ Key Laboratory of Colloid and Interface Chemistry of the Ministry of Education, School of Chemistry and Chemical Engineering, Shandong University, Jinan 250100, China.; ^4^ Department of Cardiovascular Surgery, Qilu Hospital of Shandong University, Jinan Shandong 250012, China.; ^5^ Department of Traditional Chinese Medicine, Qilu Hospital of Shandong University, Jinan Shandong 250012, China.; ^6^ Rehabilitation Center, Qilu Hospital of Shandong University, Jinan, Shandong 250012, China.; ^7^ Advanced Interdisciplinary Technology Research Center, National Innovation Institute of Defense Technology, Beijing 100071, China.

## Abstract

Osteoarthritis (OA) is a common age-related degenerative disease characterized by changes in the local tissue environment as inflammation progresses. Inspired by the wind-dispersal mechanism of dandelion seeds, this study develops responsive biomimetic microsphere–drug conjugate for OA therapy and protection. The conjugate integrates dibenzaldehyde polyethylene glycol (DFPEG) with chitosan and polyethylene glycol diacrylate (PEGDA) through dynamic covalent bonds to form a dual-network hydrogel microsphere. Based on the progression of OA, the conjugate with the surface-anchored cyclic peptide cortistatin-14 (CST-14) achieves targeted drug therapy and a self-regulating hydrogel network. In cases of progressing inflammation (pH < 5), CST-14 dissociates from the microsphere surface (viz. the drug release rate increased) and inhibits TNF-α signaling to suppress OA. Concurrently, the monomer DFPEG responsively detaches from the hydrogel network and scavenges reactive oxygen species (ROS) to protect the cartilage tissue. The ROS scavenging of DFPEG is comparable to that of coenzyme Q10 and vitamin C. The degraded PEGDA microspheres provide tissue lubrication through reused conjugates. The rat OA model successfully achieved a synergistic therapeutic effect greater than the additive effect (1 + 1 > 2). This strategy offers an approach for anchoring amine-containing drugs and has marked potential for OA treatment and protection.

## Introduction

Hydrogels are exceptional materials known for their excellent biocompatibility, allowing their various applications in biomedicine [1–4]. Microfluidic technology enables the stable production of uniform hydrogel microspheres. As a form of hydrogel, these microspheres have a higher surface-to-volume ratio and excellent injectability than bulk hydrogels [5]. Consequently, mixing microspheres with drugs is considered a more effective approach for injection therapy than injecting drugs alone, offering a new way for drug delivery [6]. Based on the combination of drugs with microspheres, if the gel network anchors the drug molecules [7], this interaction results in a prolonged drug release. This interaction can be achieved by utilizing specific chemical bonds, such as disulfide bonds or amide bonds [8,9]. However, chemical bonds that facilitate drug anchoring typically have lower bond energies, such as disulfide bonds (251 kJ/mol), which are less stable, or intermediate bond energies, such as amide bonds (305 kJ/mol), pose challenges for drug release [10]. Therefore, selecting a covalent bond with high bond energy, stability, and sensitivity to the environment, such as disease severity, is crucial for designing an intelligent drug–hydrogel delivery system.

Osteoarthritis (OA) is a global disease and has become a common cause of disability in millions of adults [11–13]. A typical feature of OA is a change in the tissue environment in the joint as the disease progresses. In various diseases related to acute and chronic inflammation, local acidification has been observed at inflamed sites [14–17]. The pH values in the inflamed areas are generally lower than those in healthy tissues, and this is related to inflammation severity and level of inflammation and disease activity [18,19]. The pH of healthy synovial fluid ranges from 7.4 to 7.8, whereas osteoarthritic joints have a lower pH between 6.6 and 7.2 [20,21]. In the inflamed cartilage region, the local pH may drop to 5 or below [22,23]. To enhance the effectiveness of medication delivery for OA therapy, a drug delivery platform that is responsive to pH can be utilized. Furthermore, a hostile microenvironment at the pathology site is primarily induced by excessive ROS and nonspecific inflammation. Lowering ROS levels in the affected area of OA can consequently reduce cellular apoptosis and enhance therapeutic efficacy.

Molecules that bind competitively to tumor necrosis factor receptors (TNFRs) have shown promise as potential treatments for inflammation by inhibiting the function of TNF-α [24,25]. CST-14 is a multi-functional neuropeptide that can be expressed in a wide variety of cells and plays a key role in many physiological and disease processes, including heart disease, immune response, and nervous system degeneration [26–29]. Recent data suggest that CST-14 exerts a protective effect in mouse models of inflammatory arthritis by countering inflammatory responses [30]. In our previous study, we observed that CST-14 has a direct binding effect on TNFRs and can act as an antagonist to suppress TNF-α–carbohydrate catabolism in vitro. In OA, CST-14 exerts an inhibitory effect on the augmented activation of the nuclear factor κB (NF-κB) signaling pathway induced by TNF-α, potentially contributing to the attenuation of cartilage degeneration [31].

So far, systemic administration and intra-articular injection of anti-inflammatory drugs are the first-line treatments for OA [32]. The therapeutic efficacy of intra-articular drug delivery depends primarily on the efficiency of the drug delivery system [33]. However, the rapid clearance of small-molecule anti-inflammatory drugs from the articular cavity and their low bioavailability limit their therapeutic efficacy [34].

In nature, dandelions exemplify a typical environmentally responsive structures [35,36]. Following fertilization and maturation, the elongated seeds are anchored to the scape. In the presence of strong winds, the seeds are dislodged from the receptacle, facilitating rapid release and enabling long-distance dispersal. Conversely, in windless or low-wind environments, dandelion seeds can maintain a relatively stable anchoring state [37]. This phenomenon helps to prevent excessive proliferation in the same location. This environmentally responsive characteristic enables dandelions to adapt to varying environmental conditions and increases the survival of their seeds [38]. Based on the insights derived from this phenomenon, an environmentally responsive biomimetic hydrogel material has been developed, which can autonomously modulate drug release rates and hydrogel network structure according to the severity of OA, enabling targeted therapy and cartilage tissue protection.

**Fig. 1.**
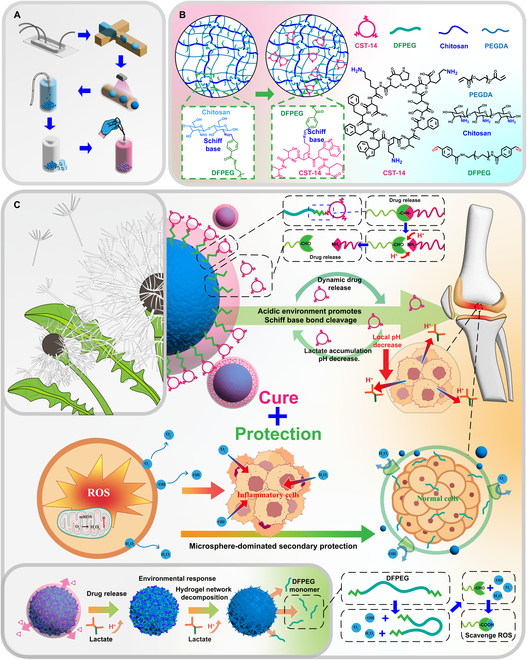
Schematic illustration of (A) the process of microsphere fabrication, (B) the binding and anchoring of CST-14 to the surface hydrogel network of microspheres, (C) the interactive dynamic treatment process of biomimetic conjugates and OA, and the protection of cartilage tissue by degraded biomimetic conjugates.

This novel hydrogel material is composed of natural monomer chitosan and dibenzaldehyde polyethylene glycol (DFPEG), forming the first network through Schiff base bonding and PEGDA forming the second network by polymerization. The precursor microspheres for the conjugates can be easily prepared using microfluidics (Fig. 1A). Excess DFPEG within the gel network results in the formation of an aldehyde-rich surface on the microspheres. The reactive aldehyde groups on the DFPEG benzene ring form Schiff base bonds with the 3 primary amines on CST-14 cyclic peptide (Fig. 1B). These bonds establish a connection between the hydrogel network and the drug and exhibit high stability under normal conditions; however, they are highly pH sensitive, enabling the construction of a dandelion-like “pappus and seed” structure. The progression of arthritis is closely associated with the pH of the joint cavity. When the condition becomes severe, a decreased pH triggers the rapid destruction of drug-anchoring structures and the first hydrogel network, resembling a “strong wind”. The “strong wind” disrupts the Schiff base bonds, dispersing the “seeds” of CST-14 and the “pappus” of DFPEG, thereby inhibiting the progression of the condition and scavenging ROS in the joint (Fig. 1C). In addition, pure PEGDA microspheres provided a lubricating function after exposure to the “strong wind” to further protect cartilage tissue. In cases where the progression of the condition is slower, these high-energy covalent bonds are far more stable than amide or disulfide bonds, allowing for prolonged drug delivery and avoiding the loss of active ingredients. Overall, this biomimetic microspheres minimized drug wastage and enhanced drug delivery efficiency by mimicking how dandelion seeds are carried by the wind. Moreover, in cases of severe inflammation, the drug delivery rate is increased by 100% or more. Furthermore, the microspheres continued to protect the cartilage after their therapeutic action by removing ROS and providing lubrication, allowing the drug delivery system to be recycled.

## Materials and Methods

### Materials

Poly (ethylene glycol) diacrylate (PEGDA, *M*_w_ = 400) was purchased from Merrier Laboratory Equipment Co. Ltd. (Shanghai, China). Span 80 was purchased from Aladdin Reagent Co. Ltd. (Shanghai, China). Chitosan (*M*_w_ = 150,000) was purchased from Shanghai Maclin Biochemical Technology Co. Ltd. (Shanghai, China). *n*-Hexadecane (purity > 99.0%) was purchased from Shanghai Ruentropy New Energy Technology Co. Ltd. (Shanghai, China). DFPEG is obtained through synthesis and subsequent purification based on the approaches described previously [39].

### Fabrication of hydrogel microspheres

Typically, Irgacure 2959 (2 wt%), glacial acetic acid (0.5 wt%), chitosan (0.5 wt%), DFPEG (1 wt%), and PEGDA (10 wt%) were dissolved in deionized water and stirred at 50 °C to obtain a homogeneous solution. Consistent with previous studies [40], the solution was then injected into a microfluidic chip of flow-focusing type to produce hydrogel microspheres. After obtaining the microspheres, they were washed 3 times with a 1:1 volume ratio of isopropanol–water solution and centrifuged to remove the oil phase. Finally, the microspheres were freeze dried and stored at −20 °C (see Fig. 1A).

### Vitro release experiments

The vitro release experiment of microspheres binding with drugs provides a better qualitative and quantitative assessment of the binding and separation process between the drug and the hydrogel matrix. In the experiment, both CST-14 solution and a control group (rhodamine B solution) were added separately to the prepared freeze-dried microspheres. The microspheres were then incubated at 2 °C for 12 h to allow the drug molecules to fully react with the gel network. The microsphere solution was added to 20 ml of phosphate-buffered saline (PBS) (pH 7 and 3.5) and placed in an ambient temperature environment. The supernatant was collected at different time intervals for analysis.

For CST-14 samples, a colorimetric assay was performed after a suitable color development process, followed by analysis using an enzyme-linked immunosorbent assay (ELISA). In contrast, rhodamine B samples were directly analyzed using a spectrophotometer.

### Numerical simulation of microfluidic droplet production

The process of droplet formation from the hydrogel precursor solution was simulated using numerical modeling. A 2-phase flow phase-field model was employed to simulate the transient behavior in a laminar flow regime. The simulation was performed with a time step of 1 ms and a total time span of 2,000 ms. The frequency of droplet generation was obtained through image acquisition and analysis. Details of simulation parameters are shown in Table S1.

### Nuclear magnetic resonance analysis

Samples 1, 2, and 3, as indicated in Table S2, were dissolved in deuterated dimethyl sulfoxide (DMSO-d6) solvent and subjected to one-dimensional proton (^1^H) spectroscopy at a magnetic field strength of 500 MHz [nuclear magnetic resonance (NMR) analysis, Ascend 500MHz spectrometer, Bruker]. The obtained data were subsequently subjected to normalization processing for further analysis. It is worth noting that CST-14 and DFPEG were dissolved in deionized water, reacted at 2 °C for 12 h, and subsequently freeze dried to obtain sample 1.

### Rheology test

To record the storage modulus (*G*′) and loss modulus (*G*″) of PEGDA–chitosan–DFPEG aqueous solution during ultraviolet (UV) curing, oscillatory frequency sweep measurements were conducted using a rheometer (MCR 302, Anton Paar, Austria) under UV irradiation at intensities of 2.47, 4.94, 9.88, and 19.76 W/cm^2^ (γ = 1%, *f* = 1 Hz). The samples used in the rheological testing are indicated in Table S3.

### Compression test

The compression tests of the hydrogels were conducted on a universal testing machine (ZLC-2D, Jinan XLC Testing Machine Co. Ltd., China) with a 2-kN load cell at a loading rate of 5 mm/min. Cylindrical hydrogel samples (9 mm in diameter, 10 mm in length) were prepared for the tests.

### Enzyme-linked immunosorbent assay

The levels of CST-14 were assayed through ELISA by using a commercial kit (Shanghai Xinyu Biotechnology Co. Ltd). The captured antibody was immobilized on an ELISA plate. The captured samples and standards containing CST-14 were incubated with a biotinylated detection antibody that binds to CST-14. The streptavidin-biotin complex (SABC) was bonded to the biotinylated detection antibody to form an immune complex. After adding the 3,3’,5,5’-tetramethylbenzidine (TMB) color development solution, a blue color appeared in the reaction wells if CST-14 was present. The addition of a stop solution turned the color into yellow. During the detection process, any unbound components are washed away. The optical density (OD) value was measured at 450 nm using an ELISA reader. The concentration of CST-14 in the sample is directly proportional to the OD value. By plotting a standard curve, CST-14 concentrations in the specimens were calculated by plotting a standard curve.

### Animals

Three-month-old Sprague–Dawley rats were purchased from Vital River Laboratory Animal Technology (Beijing, China). All animals were housed under controlled identical specific pathogen-free (SPF) standard environmental conditions (23 ± 2 °C, 12 h light/dark cycle) with free access to food and movement freedom. All animal treatments and surgical procedures were conducted in accordance with the guidelines of the Ethics Committee on Animal Experiments of Shandong University in China (approval no. 23027).

### Rat model establishment

Briefly, the rats were anesthetized with isoflurane supplied in a rat anesthesia apparatus, followed by joint surgery on the right joint by sectioning the medial meniscotibial ligament. Destabilization of the medial meniscus (DMM) surgery was performed under a microscope. A sham operation with a control rat of the same age was performed with a similar incision at the right joint capsule without a meniscal ligament section. According to the group design, 7 days after DMM surgery, 100 μl of PBS, microspheres, CST-14, and conjugate solution was injected into the knee joints of rats twice weekly. The rats were sacrificed 6 weeks after DMM.

### Human samples and ethics statement

Human cartilage specimens were obtained from 7 patients (3 males and 4 females, aged 62 to 74 years; mean age = 67.86 ± 4.14 years), who underwent total knee joint replacement surgery for OA at Qilu Hospital of Shandong University. The chondrocytes were isolated and stimulated with lipopolysaccharide (LPS) for further experiments. This study was approved by the Medical Ethical Committee of Qilu Hospital of Shandong University [approval no. KYLL-2020(KS)-311]. The patients involved in this study signed informed consent documents and voluntarily agreed to participate in this research.

### Culture of primary human cartilage cells

Briefly, cartilage slices were minced and washed clean with 1% PBS. The cells were digested with 0.2% collagenase type II (Gibco) at 37 °C for 8 h. Chondrocytes were seeded at a density of 5.7 × 10^5^ cells/cm^2^, cultured in Dulbecco’s modified Eagle’s medium (DMEM)/F12 (HyClone, Logan, USA) supplemented with 10% fetal bovine serum (FBS; Gibco, USA), 100 U/ml penicillin, and 0.1 mg/ml streptomycin (HyClone, USA), and incubated under standard conditions (37 °C, 5% CO_2_). The culture medium was replaced every 3 days.

### Biocompatibility of the biomimetic conjugates

Chondrocytes in the exponential growth phase were seeded into 96-well plates. After the treatments, the chondrocytes were incubated for 24, 48, and 72 h. A CCK-8 kit (Sigma-Aldrich, USA) was used to test the cytotoxicity. A microplate reader was used to measure the absorbance of each well at 450 nm. Similarly, the chondrocytes were incubated for 24 and 72 h. A live/dead assay kit (Beyotime, China) was used to conduct live/dead assays. Images were captured using a fluorescence microscope (Olympus IX51, Tokyo, Japan).

### MitoTracker assay

MitoTracker staining was performed to visualize the mitochondria in the primary human cartilage cells of each indicated group. The procedure was performed in accordance with the instructions of the MitoTracker Assay Kit (C1049; MitoTracker Red CMXRos, Beyotime Biotechnology) for 30 min. Then, the cells were washed and observed under a fluorescence microscope (Olympus IX51, Japan) system.

### JC-1 assay

To detect the mitochondrial membrane potential in this study, a JC-1 assay kit was used (C2006; Beyotime Biotechnology). Based on the manufacturer’s instructions, primary human cartilage cells from each indicated group in 24-well plates were stained with a JC-1 staining solution at 37 °C for 20 min while protected from light. Then, each well in the plate was washed twice with 1× JC-1 staining buffer, and the fluorescence intensity was measured using a fluorescence microscope (Olympus IX51, Japan) system. The red-to-green fluorescence ratio reflected changes in the mitochondrial membrane potential.

### Radiographic assessment

Radiology (GE XR650, USA) was performed to assess the degree of joint degeneration. Digital images were obtained using a radiographic plate system included with the instrument. The knee-space width of the different rat groups were analyzed based on the scanned images.

The scanning protocol included an isometric resolution of 15 μm, with radiology energy settings of 70 kV and 200 μA. The microstructure of the knee was measured using a Scanco μCT50 scanner (Scanco Medical AG, Bassersdorf, Switzerland). Prior to histological processing, samples were fixed in paraformaldehyde and used for micro-computed tomography (CT). The scanned images from each group were evaluated at the same threshold to allow the 3-dimensional structural reconstruction of each sample.

### Histological analysis

The collected knee specimens were fixed in 4% paraformaldehyde followed by paraffin embedding. The tissue sections were mounted on glass slides using a microtome (Leica, Germany). The Safranin O–Fast Green assay was performed according to standard protocols. The sections were stained with a Safranin O staining kit (G1371, Solarbio) according to the manufacturer’s recommended procedure.

### Immunohistochemistry

The immunohistochemical evaluation was conducted using an Immunohistochemistry Kit (SP-9000, ZSGB-BIO, China) following the manufacturer’s instructions. The tissue sections were dewaxed and hydrated, followed by deparaffinization and incubation in 3% H_2_O_2_. Antigen retrieval was performed using tris-ethylenediaminetetraacetic acid antigen retrieval buffer (C1038, Solarbio) at 95 °C for 10 min, and endogenous peroxidase was blocked for 10 min. Subsequently, the sections were blocked with goat serum at room temperature for 30 min and incubated overnight at 4 °C with primary antibodies against aggrecan (1:200, Abcam) and matrix metalloproteinase-13 (MMP-13) (1:200, Abcam). Next, the sections were incubated with secondary antibodies, goat anti-rabbit immunoglobulin G (IgG), for 1 hour at room temperature, followed by 15 min of incubation with horseradish peroxidase (HRP)-labeled streptomyces ovalbumin. The sections were rinsed with phosphate-buffered saline containing Tween-20 (PBST) and visualized using a DAB kit (ZSGB-BIO, China). Subsequently, the slices were washed with tap water, counterstained with hematoxylin, dehydrated, and coverslipped with neutral resin. Image acquisition was performed using a panoramic digital slice scanning microscope (Olympus, Tokyo, Japan), and immunopositivity within the fields was calculated using ImageJ software (NIH, MA, USA).

### Real-time PCR

Total mRNA was extracted from chondrocytes using RNA Fast 200 (Fastagen, Shanghai, China). Purified RNA was reverse transcribed into cDNA using qPCR-RT Master Mix (Toyobo, Japan). Finally, a real-time quantitative analysis of the genes was performed with 2× SYBR Green qPCR Master Mix (APExBIO, Houston, USA). Relative mRNA expression levels were normalized to an internal control and calculated according to the 2^−ΔΔCt^ method. Glyceraldehyde-3-phosphate dehydrogenase (GAPDH) was used as an internal control.

### Western blot analysis

RIPA Lysis Buffer (APExBIO, Houston, USA) was used to lyse the chondrocytes. Then, the solution was centrifuged at 12,000 rpm at 4 °C for 15 min. Sodium dodecyl sulfate–polyacrylamide gel electrophoresis (SDS-PAGE) was used to separate proteins at 80 V. Then proteins were transferred onto 0.22-μm polyvinylidene fluoride membranes (Millipore, USA) at 260 mA. QuickBlock blocking buffer (Beyotime, China) was used to block the membranes at room temperature for 20 min. Then, the membranes were treated with the following primary antibodies at 4 °C overnight: MMP-13 (1:1,000, Abcam), ADAMTS-5 (1:1,000; Abcam), Bax (1:1,000, Abcam), cleaved caspase-3 (1:1,000, Cell Signaling Technology), and β-actin (1:1,000, Cell Signaling Technology). Finally, the membranes were incubated with a secondary antibody (1:2,000, Proteintech) at room temperature for 1 h. An enhanced chemiluminescence kit (Millipore, USA) was applied to detect the membranes. Protein contents were quantified by ImageJ software.

### Immunofluorescence staining

Chondrocytes were cultured in 24-well plates at a density of 2 × 10^4^ cells per well. After being incubated with various treatments, the cells were fixed and incubated with the primary antibodies MMP-13 (1:100), ADAMTS-5 (1:100), iNOS (1:100), and cyclooxygenase-2 (COX-2) (1:100) overnight at 4 °C. Afterward, the cells were incubated with secondary antibodies for 1 h. After being stained with 4′,6-diamidino-2-phenylindole (DAPI) for 5 min, the images were acquired using a fluorescence microscope (Olympus IX51, Japan). The mean fluorescence intensity was calculated based on ImageJ software.

### DPPH radical scavenging test

1,1-Diphenyl-2-picryl-hydrazyl radical (DPPH) was dissolved in anhydrous ethanol to prepare a solution with a concentration of 0.2 mg/ml. In a 20-ml sample vial, 5 ml of hydrogel precursor solution was added and exposed to UV light for 5 min for curing. Subsequently, 5 ml of deionized water and 5 ml of the DPPH solution were separately added. The vials were shaken at 0-, 24-, 48-, and 72-h time points, and the supernatant was collected for photography and absorbance measurements using a spectrophotometer.

### Intracellular ROS detection

Chondrocytes were cultured in a 6-well plate at a density of 2 × 10^3^ cells per well. Subsequently, different groups were treated accordingly. Briefly, after stimulation with 1 μl/ml of 3% H_2_O_2_, DFPEG with the same molar mass as vitamin C (0.1 mM) or coenzyme Q10 (0.16 mM) was added. After incubating for 72 h, chondrocytes were washed 3 times and then incubated with 2,7-dichlorofluorescein diacetate (DCFH-DA) (Beyotime, China) at 37 °C for 30 min. Subsequently, chondrocytes were washed 3 times with PBS, and the levels of ROS were measured using a fluorescence microscope (Leica, Germany).

### Statistical analysis

The data were presented as mean ± standard deviation (SD). Statistical analysis was performed with the *t* test and one-way analysis of variance (ANOVA) analysis (SPSS software, version 26.0, SPSS Inc.). *P* < 0.05 was considered as statistically significant.

## Results

### Photopolymerization and mechanical properties of microspheres

**Fig. 2.**
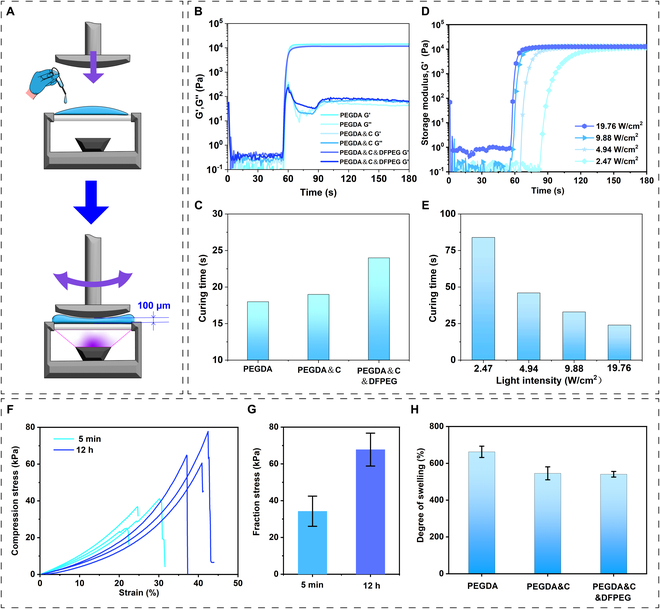
The rheological and mechanical properties of the hydrogel materials used in biomimetic conjugates. (A) Schematic diagram of sample testing in a rheometer. (B) Amplitude scanning test at 1 Hz and 1% strain under UV irradiation (250 to 450 nm, 19.76 W/cm^2^). (C) Curing time comparison bar chart for different hydrogel precursors. (D) Amplitude scanning test at 1 Hz and 1% strain under UV irradiation (250 to 450 nm, 2.47, 4.94, 9.88, and 19.76 W/cm^2^). (E) Curing time comparison bar chart for hydrogel precursors under varying UV intensities. (F) Compression stress–strain curves and (G) maximum compression stresses of the PEGDA&C&DFPEG hydrogel after being immersed in deionized water for 5 min and 12 h. The compression tests were repeated 3 times to check the reproducibility. (H) Degree of swelling of the hydrogels (PEGDA, PEGDA&C, PEGDA&C&DFPEG).

The rheometer rotor is used to compress the samples to a thickness of 100 μm to better simulate the actual situation in microfluidic experiments (Fig. 2A). By performing rheological tests on pure PEGDA, chitosan, PEGDA and chitosan (PEGDA&C), and chitosan with PEGDA and DFPEG (PEGDA&C&DFPEG), it was observed that their storage modulus and shear modulus exhibited similar changes under mercury lamp irradiation at 19.76 W/cm^2^ (Fig. 2B). To better quantify the curing time of the gel, the following Eq. 1 was introduced [40].

$$T_{c}=\frac{G_{i^{\prime }}}{G_{{i+1}^{\prime }}}-1$$ (1)

where *T_c_* is the growth rate of storage modulus for the aqueous phase solution to cure into a hydrogel, $G_{i}^{\prime }\;$ is the storage modulus recorded at a point in time, and $G_{i+1}^{\prime }$ is the storage modulus recorded at the next point in time. When *T_c_* is consistently less than 1%, indicating that the storage modulus is no longer undergoing drastic changes, the hydrogel is considered to have completed solidification.

By utilizing Eq. 1 to analyze the rheological curve in Fig. 2B, the exact curing time shown in Fig. 2C can be obtained. The chitosan and DFPEG network affect the formation of the PEGDA network. Nonetheless, the biomimetic microspheres’ hydrogel system achieves complete curing within 30 s, demonstrating excellent photopolymerization performance for the microsphere production with microfluidic chips. In addition, Fig. 2D and E shows the rheological analysis of the curing performance of the biomimetic conjugate’s hydrogel under different UV light intensities. It was observed that at UV intensities above 9.88 W/cm^2^, rapid curing of approximately 30 s could be achieved. Therefore, when preparing biomimetic microspheres, it is advisable to select a UV intensity above 9.88 W/cm^2^ to ensure thorough curing and avoid insufficient crosslinking of PEGDA monomers, which may lead to additional toxicity. Figure 2F shows the compressive stress–strain curves of the PEGDA&C&DFPEG hydrogel after being immersed in deionized water for 5 min and 12 h. The fracture stress increased from 34.3 ± 8.2 kPa to 67.8 ± 8.9 kPa (Fig. 2G), and the fracture strain increased from 25.5 ± 4.1% to 41.1 ± 2.9% (Fig. S1) when the immersed time extended from 5 min to 12 h. The results demonstrate that the mechanical properties of the PEGDA&C&DFPEG hydrogel cannot be diminished under aqueous condition. Additionally, the biomimetic microspheres made of dual-network hydrogels exhibit lower swelling ratios compared to pure PEGDA network hydrogels (Fig. 2H).

### The process of fabricating microspheres and their characteristics

**Fig. 3.**
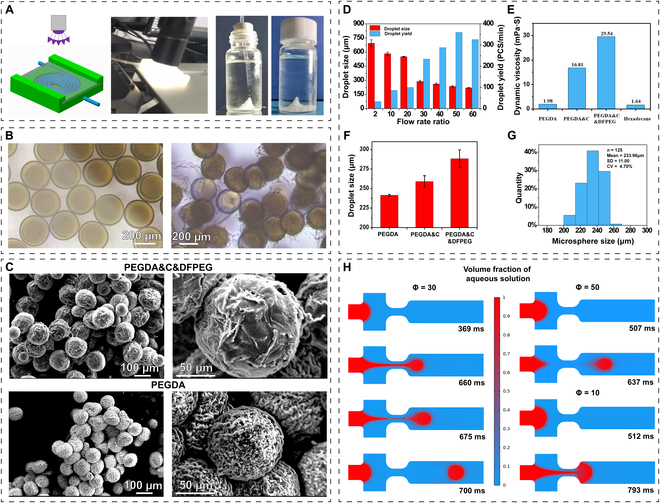
Images of microspheres and their preparation process, as well as their characteristics and numerical simulation. (A) Practical production process of transforming gel precursor droplets into hydrogel microspheres through photocuring. (B) Under an optical microscope, the freshly produced hydrogel microspheres (left) and the rehydrated hydrogel microspheres after freeze-drying (right). (C) SEM image of dandelion-inspired biomimetic dual-network microspheres and microspheres made of pure PEGDA with a single network structure. (D) Size and yield of droplets produced using a microfluidic chip at different flow rate ratios. (E) The viscosity of hydrogel precursor solutions varies with different components. (F) Droplet size of hydrogel precursor solutions with different compositions during production in a microfluidic chip. (G) Size of microspheres obtained after the solidification of dandelion-inspired biomimetic dual-network gel precursor droplets. (H) Numerical simulation of the droplet generation process.

As shown in Fig. 3A, the droplets to be solidified are passed through a helical-shaped channel, allowing them to be adequately exposed to UV radiation in the UV region. This process ensures the complete initiation of PEGDA, leading to the formation of the second network. Subsequently, the fully solidified microspheres are collected in a glass vial. The freshly collected microspheres, as shown in the left image of Fig. 3B, exhibit a perfect spherical shape. After undergoing complete freeze-drying and subsequent rehydration, a pH change triggers a phase transition in the hydrogel network, resulting in the formation of linear structures, as depicted in the right image of Fig. 3B [41]. This phenomenon further facilitates drug anchoring, as the phase-transitioned chitosan, along with DFPEG, reassembles into a microgel network on the surface of the microspheres, where they can effectively load and anchor drugs. The microgel network formed on the surface of these microspheres not only achieves functional similarity to a dandelion but also exhibits a similar structure.

The upper image in Fig. 3C depicts the morphology of these biomimetic microspheres after freeze-drying, revealing the absence of a prominent porous structure. This phenomenon can be attributed primarily to the formation of a dense network structure through the Schiff base bonds between chitosan and DFPEG, as well as the polymerization of PEGDA. The lower image in Fig. 3C represents hydrogel microspheres prepared from a pure PEGDA solution with the same precursor solution content as employed in this study. In comparison with the upper image, it is evident that the pure PEGDA microspheres exhibit a distinct porous structure and smaller size. These observations substantiate the presence of a dual-network structure in these biomimetic microspheres.

In addition to examining and analyzing the morphological characteristics of the microspheres, the regularities of the hydrogel microsphere preparation process can be obtained by comparing the properties of different gel precursor solutions. By controlling the flow rate ratio within the microfluidic chip, different sizes of the droplets can be rapidly obtained, thereby achieving hydrogel microspheres with varying injectability and specific surface area (Fig. 3D). This result enables customized treatments tailored to the specific needs of individual patients. Equation 2 is introduced to describe the flow rate ratio:

$$\Phi =\frac{Q_{O}}{Q_{A}}$$ (2)

where *Φ* is the flow rate ratio, *Q_o_* is the flow rate of the oil phase solution, and *Q_A_* is the flow rate of the aqueous phase solution. By adjusting the *Φ* from 2 to 60, it is possible to achieve a wide range of microsphere sizes.

The solution containing DFPEG, chitosan, and PEGDA exhibits the highest viscosity compared to other gel precursor solutions (Fig. 3E). This is primarily attributed to the partial crosslinking of chitosan and DFPEG through dynamic covalent bonds before the formation of the second network by PEGDA. This observation indirectly confirms the presence of a dual hydrogel network in these biomimetic microspheres. The hydrogel precursor solution with high viscosity also brings additional characteristics. In previous studies, it was observed that high-viscosity solutions, without additional compensation for water phase flow rates, tend to experience greater loss of kinetic energy before entering the chip [40]. As a result, the droplet formation rate slows down, leading to the generation of larger droplets. As shown in Fig. 3F and G, the microsphere precursor solution (PEGDA&C&DFPEG) possesses larger droplet sizes, thereby obtaining microspheres with larger volumes. This phenomenon can be observed in the scanning electron microscopy (SEM) images, where the biomimetic microspheres exhibit a significantly larger volume compared to the pure PEGDA microspheres, even under the same Φ.

By utilizing experimental data and conducting numerical simulations, the entire process of droplet production in the formation of biomimetic microspheres was simulated. As shown in Fig. 3H, adjusting to higher flow rate ratios resulted in the formation of smaller and more uniform microspheres. Specifically, under a flow rate ratio of 50, the droplet generation frequency was higher than that under flow rate ratios of 10 and 30, resulting in smaller droplet volumes. Conversely, reducing the shear rate of the oil phase over the pre-polymer solution in the water phase resulted in larger droplets, which led to the production of microspheres with a larger diameter. Understanding this process allows us to adjust the size of microspheres based on the treatment needs of patients, thereby controlling the specific surface area and achieving the ability to freely modify drug loading capacity.

### The environmental responsiveness of conjugates

**Fig. 4.**
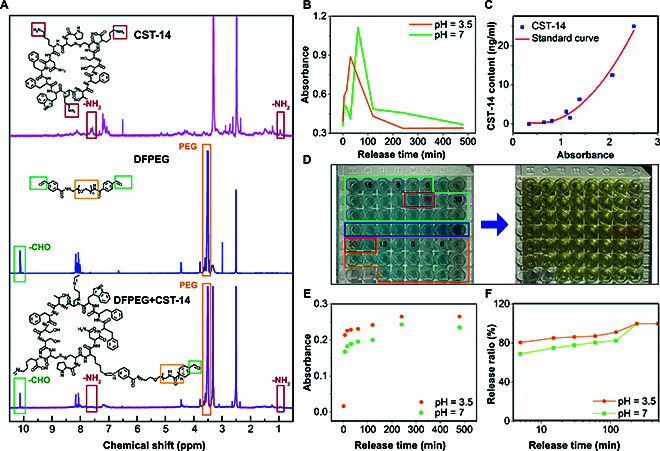
In vitro release and NMR spectroscopy of biomimetic conjugates. (A) The one-dimensional proton NMR spectra were obtained for DFPEG, CST-14, and their complex. The CST-14 spectrum is represented by the pink curve, the DFPEG spectrum is represented by the blue curve, and the reactants of both compounds are represented by the purple curve. (B) Release curves of biomimetic conjugates loaded with CST-14 in PBS buffer solutions at pH 3.5 and pH 7. (C) Standard curve of CST-14. (D) ELISA colorimetric images of the in vitro release of CST-14 from biomimetic conjugates. (E) Absorbance curve of the release of rhodamine B from biomimetic microspheres. (F) Release rate curve of rhodamine B from biomimetic microspheres.

NMR hydrogen spectroscopy analysis was conducted to provide evidence of the anchoring of CST-14 to the hydrogel network following its reaction with DFPEG. Figure 4A illustrates the results of the one-dimensional hydrogen spectroscopy analysis of CST-14, DFPEG, and their reaction products. In the pink curve, the red box [0.97 parts per million (ppm) and 7.60 ppm] highlights the peak corresponding to the amino group (^2^H) in the CST-14 molecule. The green box (10.12 ppm) in the blue curve indicates the peak corresponding to the aldehyde group (^1^H) in the DFPEG molecule, while the yellow box (3.51 ppm) represents the hydrogen (^1^H) signals from the polyethylene glycol portion of DFPEG. Upon comparative analysis of the 3 curves, a notable reduction in the peak associated with the amino group is observed following the reaction between CST-14 and an excess of DFPEG. Additionally, by comparing the intensity ratio of the aldehyde group hydrogen peak to the polyethylene glycol hydrogen peak before and after the reaction (decreasing from 9.00 ×10^−3^ to 5.93×10^−3^), a decrease of 34% is observed. This indicates that the aldehyde groups in DFPEG participate in the reaction and are consequently consumed. These results provide evidence confirming the occurrence of the reaction between CST-14 and DFPEG, leading to the anchoring of CST-14 within the hydrogel network. The original one-dimensional proton NMR spectrum of the sample is provided in the Supplementary Materials.

Anchoring CST-14 to the hydrogel network is the foundation for creating a dandelion-like structure. However, it is crucial to dynamically regulate the release of carried drugs based on environmental conditions, such as the severity and urgency of arthritis. At pH 3.5, the concentration of CST-14 in the supernatant reaches its peak at 30 min, whereas at pH 7, it takes twice as long (Fig. 4B). The above evidence demonstrates the ability of these bioinspired conjugates to dynamically adjust drug release based on environmental conditions. In addition, CST-14, being a cyclic peptide, is more susceptible to hydrolysis under acidic conditions (pH 3.5) compared to a normal environment (pH 7). As a result, the samples in the pH 3.5 buffer solution exhibit relatively lower final released concentrations and faster restoration to the initial levels in subsequent time points [42].

Figure 4C presents the empirical standard curve (Eq. 3) for measuring the CST-14 solution concentration, facilitating the determination of its concentration in vitro release experiments.

$$Y=1\;.84-6\;.34\;x+6\;.05\;x^{2}$$ (3)

where the *y* axis is the concentration of CST-14 in the solution, while the *x* axis is the absorbance of the solution. By comparing the standard curve with the corresponding release curve under pH 7 normal environment, it can be observed that the long-term release of CST-14 is adequately ensured (release time greater than or equal to 8 h).

Figure 4D displays the samples tested in the environment-responsive in vitro release experiment using the ELISA assay. The blue-colored liquid represents the sample after the addition of the TMB color reagent, while the yellow-colored liquid indicates the samples after the addition of the termination solution. Within the blue samples, the green boxes from right to left represent the sample liquids obtained at 5/15/30/60/120/240/480 min under pH 7 (2 sample liquids for each time point). The yellow box represents the sample liquid under pH 3.5, while the blue box represents the sample liquids used for the standard curve, with decreasing concentrations from right to left. Similarly, the colorimetric results of the sample liquids indicate that under the context of severe OA, the Schiff base bonds between the drug and DFPEG are disrupted, allowing CST-14 to quickly detach from the hydrogel network, achieving precise release.

In addition to the tests conducted to evaluate the anchoring, dynamic modulation, and environment-responsive behavior between CST-14 and the hydrogel network, in vitro release experiments were performed using rhodamine B to demonstrate the versatility of these bioinspired microspheres in drug loading. Figure 4E and F represents the release amount and release rate of rhodamine B from the microspheres under different environments, respectively. Both figures indicate a potential environmentally responsive release trend between the drug molecules and the hydrogel network. This phenomenon may be attributed to the fact that, in addition to anchoring, a portion of the drug molecules is adsorbed within the hydrogel network. However, the acidic environment also disrupts the Schiff base network between chitosan and DFPEG, converting the dual-network microspheres into single-network microspheres, thereby facilitating the release of drug molecules.

### Biocompatibility of the biomimetic conjugates

**Fig. 5.**
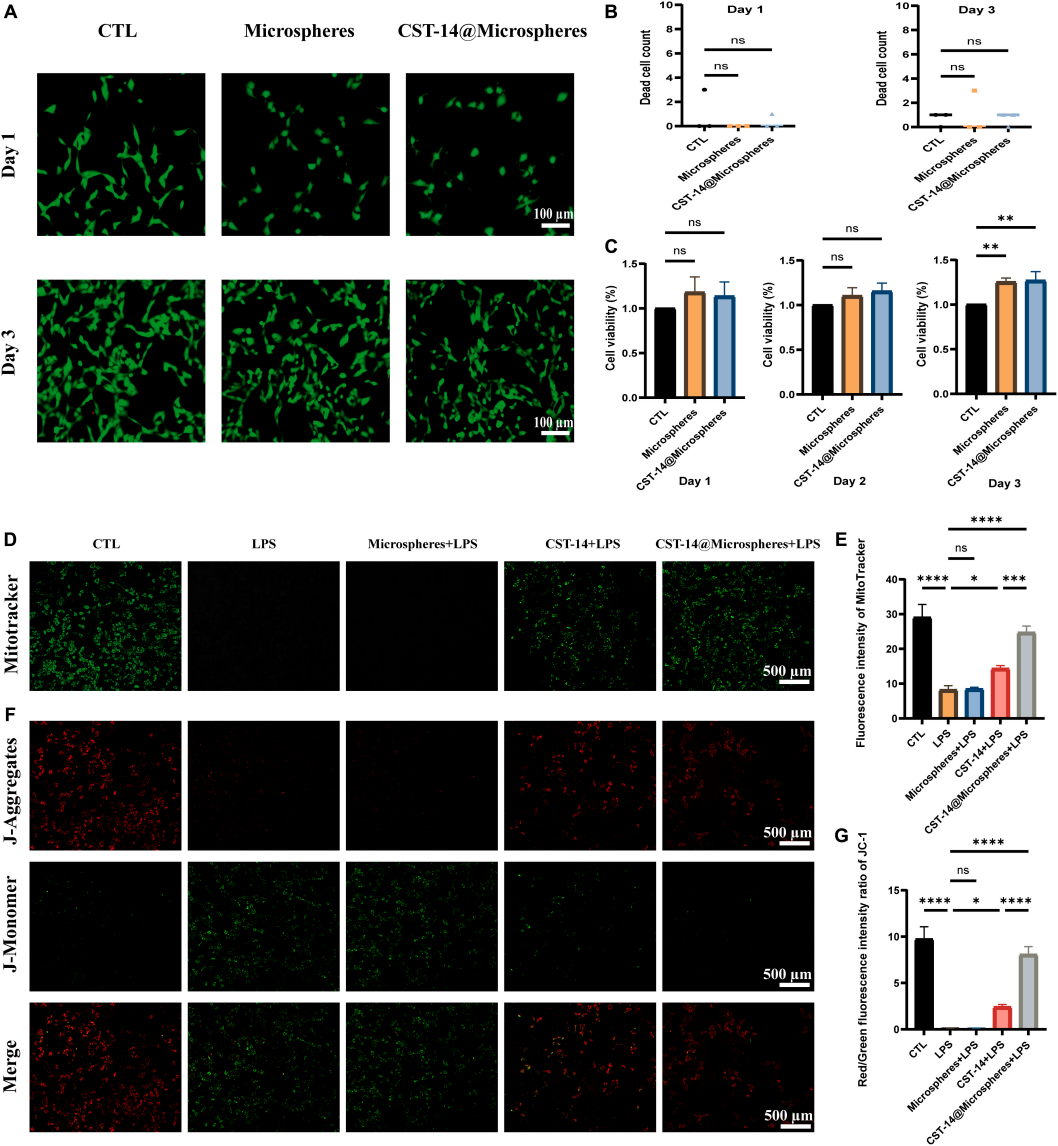
Biocompatibility and mitochondria protection of biomimetic conjugates. (A) Calcein-AM (green)/propidium iodide (red) staining of chondrocytes after incubation with various treatments. (B) Number of dead cells in different treatments. (C) Cell viability of chondrocytes after different treatments. (D) The mitochondrial function of chondrocytes was detected by MitoTracker. (E) Relative quantification of MitoTracker fluorescence. (F) Mitochondrial membrane potential was detected by JC-1 assay. (G) Relative quantification of JC-1 fluorescence. Error bar represents mean ± SD. ***P < 0.001, **P < 0.01, *P < 0.05.

To investigate the biological effects of the microspheres and the biomimetic conjugates in vitro, chondrocytes were cultured with media containing the microspheres and the biomimetic conjugates to assess any potential cytotoxicity. The results show that the number of cells does not decrease after 1 and 3 days of incubation with biomimetic conjugates (Fig. 5A and B). At the same time, we used CCK-8 to detect cell viability, and the cell viability is assessed by measuring absorbance changes (Tables S4 to S6). After incubation with microspheres and biomimetic conjugates for 1, 2, and 3 days, cell viability was not affected. The cell viability rates among the 3 groups showed no significant difference on the first and second days. However, on the third day, the cell viability rates in the microspheres group and biomimetic conjugates group are 1.25 and 1.27 times higher, respectively, compared to the control group (Fig. 5C). The results show that biomimetic conjugates have good biocompatibility in vitro.

### Biomimetic conjugates reversed mitochondrial damage in chondrocytes

The mitochondrial membrane potential of chondrocytes, measured by MitoTracker staining, showed that LPS interfered with mitochondrial function. The microspheres + LPS group did not improve mitochondrial function, while the CST-14 + LPS group and biomimetic conjugates + LPS group showed improved mitochondrial function. The fluorescence intensities of the CST-14 + LPS group and biomimetic conjugates + LPS group were 1.74 and 3.00 times higher than the LPS group, respectively. More importantly, the biomimetic conjugates + LPS group almost reverses this trend under LPS stimulation compared to the CST-14 + LPS group (Fig. 5D and E). Furthermore, the JC-1 assay confirmed that the biomimetic conjugates + LPS group had significantly improved mitochondrial function in the presence of LPS compared to the CST-14 + LPS group (Fig. 5F and G).

### Radiographic evaluation of OA

**Fig. 6.**
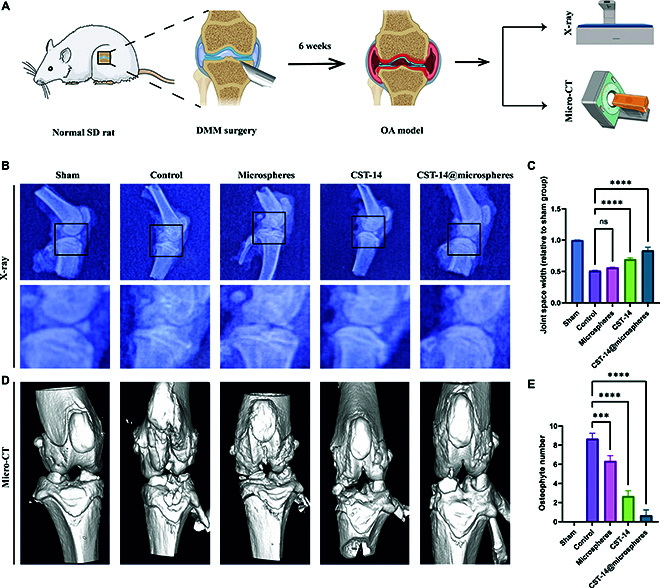
Biomimetic conjugates can delay joint space narrowing and subchondral bone changes in OA rats. (A) Model of operation and the sketch of radiological examination. (B) Representative x-ray image of joint space width. (C) The relative joint space width was measured from x-ray images. (D) Osteophyte formation and ectopic subchondral sclerosis were detected by micro-CT scanning. (E) Osteophyte number assay based on micro-CT. Error bar represents mean ± SD. ***P < 0.001, **P < 0.01, *P < 0.05.

The rats were sacrificed and performed 6 weeks after the DMM OA-induced surgery. The knee joint was then radiographed and scanned with a micro-CT system (Fig. 6A). The joint space width was first evaluated with radiographs in 5 groups of rats. The results showed that the joint space width of the 4 groups is different. The width of the joint gap was narrowest in the control group, followed by the microspheres group and CST-14 group. The joint space width in the CST-14 group was 1.36 times wider than that of the control group. However, there were no significant differences in joint space width between the microspheres group and the control group. The biomimetic conjugates group has the widest joint space width in OA. The joint space width in the biomimetic conjugates group is 1.64 times wider than that of the control group. The sham group has a standard joint space width and served as a negative control (Fig. 6B and C). However, joint space width was insensitive to assessment of mild and moderate OA [43]. In order to better evaluate the treatment effect of OA, we further analyzed the changes of knee joint and subchondral bone by micro-CT. In the reconstructed images, the biomimetic conjugates group showed better joint morphology, fewer bone fragments, and reduced osteophyte formation in the DMM model knee joints (Fig. 6D and E). Radiology and micro-CT results showed that the 3 treatment groups were effective in slowing the progression of OA. However, the biomimetic conjugates combine well the therapeutic effect of CST-14 with the drug sustained delivery and the joint lubrication ability of microspheres. Therefore, biomimetic conjugates can play a better role in alleviating cartilage aging in the DMM OA model.

### Histological analysis of OA

**Fig. 7.**
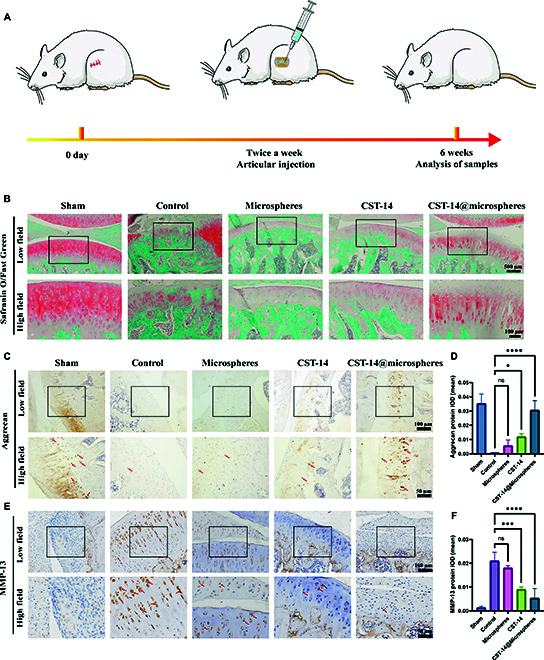
Biomimetic conjugates promote cartilage repair in OA rats. (A) Schematic diagram of the intra-articular drug administration process in rats. (B) Representative images of Safranin O–Fast Green staining. (C) Immunohistochemical (IHC) analysis of aggrecan in the knee joint in different groups (IHC positive: brown). (D) Quantitative analysis of aggrecan in various treatments. (E) IHC analysis of MMP-13 in the knee joint in different groups (IHC positive: brown). (F) Quantitative analysis of MMP-13 in various treatments. The black box represents the high-magnification field of view. Error bar represents mean ± SD. ***P < 0.001, **P < 0.01, *P < 0.05.

After completion of DMM modeling surgery, the rats were treated twice weekly with intra-articular drug injections. After 6 weeks, the rats were euthanized for histological analysis of the tissues (Fig. 7A). To further investigate the inhibitory effect of biomimetic conjugates on articular cartilage degeneration, Safranin O–Fast Green staining and immunohistochemistry were performed on articular specimens at 6 weeks after surgery. Safranin O–Fast Green staining showed a decrease in proteoglycans in the control group. There were some signs of injury repair in the microspheres group and CST-14 group, but the biomimetic conjugates group showed substantial improvement. The erosion of articular cartilage, such as cracks and deformation, was most obvious in the control group, followed by the microspheres group and CST-14 group. The biomimetic conjugates group exhibited better morphological integrity and reduced erosion of cartilage surface destruction, most similar to the sham group (Fig. 7B). The results showed that biomimetic conjugates could effectively maintain the thickness of articular cartilage. At the same time, the protein expression levels of 2 important articular cartilage biomarkers were measured (Fig. 7C and E). The results showed that compared with the control group, there were no statistically significant difference in the expression of aggrecan and MMP-13 proteins in the microspheres group. Compared with the control group, the expression of aggrecan protein was increased and the expression of MMP-13 protein was decreased in the CST-14 group and biomimetic conjugates group. At the same time, aggrecan and MMP-13 protein expression in the biomimetic conjugates group showed the most obvious changes. The integrated OD of aggrecan protein was increased by 46.49 times compared to the control group, while the integrated OD of MMP-13 protein was reduced by 3.94 times compared to the control group (Fig. 7D and F). These results suggest that biomimetic conjugates can improve the cartilage delivery efficiency of therapeutic drug CST-14. Biomimetic conjugates deliver drugs to cells effectively after intra-articular injection and are more effective in protecting chondrocytes from aging than injected CST-14, providing an attractive method for OA treatment.

### Biomimetic conjugates retard cartilage degradation by improving inflammation and metabolic disorders and reducing apoptosis in chondrocytes

**Fig. 8.**
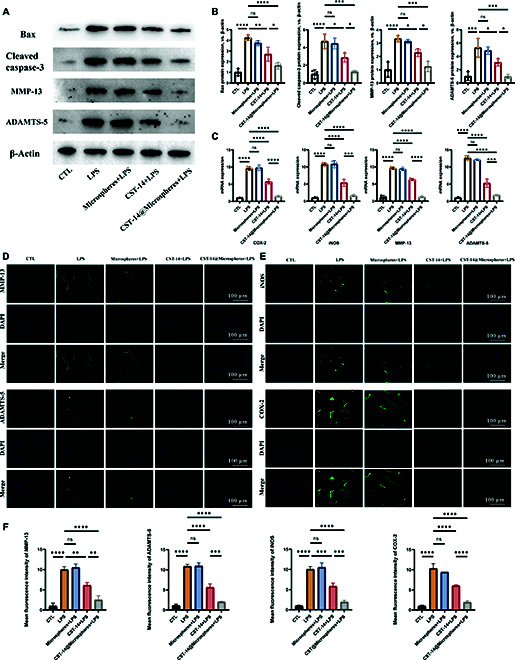
Biomimetic conjugates can reduce chondrocyte inflammation and apoptosis. (A) Expression levels of apoptosis and inflammation-related proteins on chondrocytes after different treatments. (B) Quantification of related proteins was calculated by ImageJ. (C) Reverse transcription PCR results of mRNA corresponding to COX-2, iNOS, MMP-13, and ADAMTS-5 in chondrocytes (value of *y* axis: 2^−ΔΔCT^). (D) Immunofluorescence images of MMP-13 and ADAMTS-5 (green) in different groups of chondrocytes. (E) Immunofluorescence images of iNOS and COX-2 (green) in different groups of chondrocytes. (F) Quantitative immunofluorescence analysis was obtained from (D) and (E). Error bar represents mean ± SD. ***P < 0.001, **P < 0.01, *P < 0.05.

In this study, chondrocytes were stimulated with LPS in the presence of PBS, microspheres, CST-14, and biomimetic conjugates. After 5 days, biomarkers of inflammation and apoptosis were detected by Western blot (Fig. 8A and B), real-time polymerase chain reaction (PCR) (Fig. 8C), and immunofluorescence (Fig. 8D to F). As a result, the gene expression levels and protein level of pro-inflammatory factors, including Bax, cleaved caspase-3, MMP-13, ADAMTS-5, COX-2, and inducible nitric oxide synthase (iNOS), are enhanced by LPS. The increased biomarker levels were reversed in the Microsphere, CST-14, and biomimetic conjugates groups. Moreover, the biomimetic conjugates group showed a significantly reversed increase with a more pronounced effect than that of the CST-14 group. The gene expression levels of MMP-13, ADAMTS-5, COX-2, and iNOS in the biomimetic conjugates group were decreased by 7.42, 6.84, 6.21, and 6.66 times, respectively, compared to the LPS group. The protein expression levels of Bax, cleaved caspase-3, MMP-13, and ADAMTS-5 in the biomimetic conjugates group are reduced by 2.61, 3.72, 2.69, and 5.56 times, respectively, compared to the LPS group. Immunofluorescence assays for MMP-13, ADAMTS-5, iNOS, and COX-2 also showed similar results, indicating that the biomimetic conjugates significantly inhibited matrix degradation and the inflammatory response of chondrocytes. These results suggest that LPS stimulation increases the levels of these biomarkers and that the biomimetic conjugates are most resistant to LPS-induced chondrocyte apoptosis and inflammation.

### DFPEG suppresses H_2_O_2_-induced mitochondrial ROS and oxidative stress in chondrocytes

**Fig. 9.**
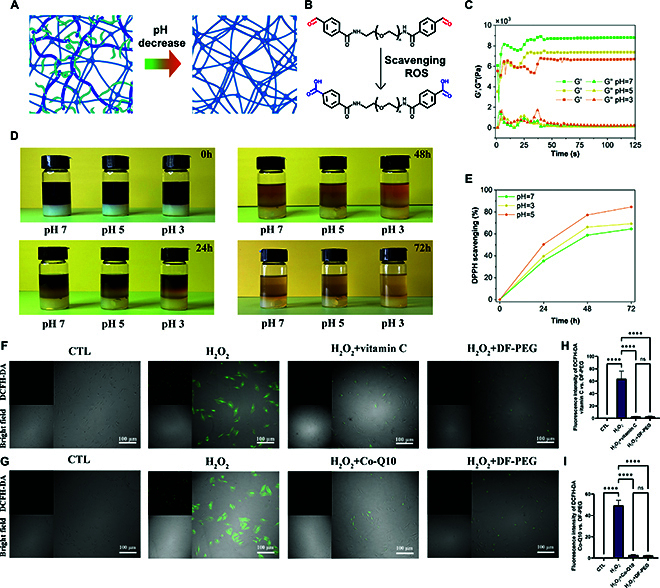
The ROS scavenging effect of the composite after degradation. Schematic diagram of (A) hydrogel network degradation and (B) DFPEG oxidation. (C) Rheological testing of gel network degradation. (D) DPPH free radical scavenger test of hydrogel network. (E) Efficiency of DPPH scavenging through the hydrogel network under different acidity conditions. (F and G) Intracellular ROS detection by DCFH-DA. (H and I) Quantification of relative ROS level. Error bar represents mean ± SD. ***P < 0.001, **P < 0.01, *P < 0.05.

After drug release, the hydrogel network of the conjugate continued to degrade in the acidic environment, releasing the DFPEG monomer (Fig. 9A). As shown in Fig. 9B, the highly reactive aldehyde groups on DFPEG absorbed ROS and were oxidized to carboxyl groups. Rheological tests of the hydrogel material confirmed the degradation of the network, with the modulus of the material gradually decreasing with increasing acidity (Fig. 9D). Experiments were conducted to further validate the scavenging action of DFPEG within the hydrogel network through the DPPH radical scavenging device. As shown in Fig. 9C, the acidic environment significantly induced the degradation of the gel network and increased the ROS scavenging efficiency. Figure 9E shows the DPPH scavenging efficiency of the gel material at different time points.

The cellular antioxidant activity of DFPEG was assessed using a DCFH-DA probe to measure the intracellular ROS levels. It was observed that H_2_O_2_ typically induces an increase in ROS levels in chondrocytes, while treatment with DFPEG resulted in a reduction of ROS levels (Fig. 9F and G). Compared with H_2_O_2_ induction alone, the addition of DFPEG caused a 4.9-fold decrease in the intracellular ROS levels, demonstrating that the antioxidant capacity of DFPEG was comparable to that of vitamin C and coenzyme Q10 (Fig. 9H and I). These results indicate that DFPEG enhanced the antioxidant stress response in chondrocytes.

## Discussion

The utilization of hydrogel microspheres for drug loading and various therapeutic applications has been widely reported in previous studies [44–46]. Based on this premise, drug–hydrogel network anchoring systems utilizing disulfide and amide bond linkages have also been developed. However, it should be noted that the relatively low bonding energies of disulfide bonds (~251 kJ/mol) and amide bonds (~305 kJ/mol) make them susceptible to environmental perturbations and potential breakdown, leading to drug detachment from the hydrogel network and a decrease in the drug loading capacity of the hydrogel–drug system [10]. The Schiff base bond, as a C═N double bond, possesses a markedly high bond energy (~615 kJ/mol), which is twice that of the previously mentioned bonds. This characteristic imparts exceptional stability to the Schiff base bond under normal environmental conditions, enabling long-lasting drug loading within the hydrogel system. Additionally, the Schiff base bond exhibits high pH sensitivity and reactivity, allowing for swift capture of drug molecules and their anchoring within the hydrogel network while facilitating decomposition in acidic environments [47].

In the dandelion-inspired biomimetic conjugates developed for the treatment of OA, highly active and biocompatible DFPEG was used as the “pappus” of the dandelion structure to trap and anchor the drug. The first layer of the hydrogel network was formed via a Schiff base reaction between chitosan and DFPEG. Subsequently, the second layer of the gel network was constructed with PEGDA by photopolymerization with the assistance of a microfluidic chip and UV irradiation [48]. This process resulted in the formation of the “dandelion core”. CST-14 is a cyclic peptide with 3 active amino groups that exhibit therapeutic effects on OA, which can protect chondrocytes in an inflammatory environment, can reduce abnormal remodeling of the subchondral bone, and has great potential in the treatment of OA (Fig. 1B). The ability of CST-14 to delay cartilage degeneration is related to its dose, and maintaining a sufficient CST-14 concentration for a long time can effectively slow the progression of OA.

The active amino groups of CST-14 were rapidly captured and anchored by DFPEG within the gel network, thus achieving the complete construction of the dandelion structure. The mechanism of hydrogel–drug anchoring and the molecular formulas of CST-14, DFPEG, PEGDA, and chitosan involved in the reactions are depicted in Fig. 1B. The process of preparing hydrogel microspheres using a microfluidic chip involves the formation of microdroplets within the chip, followed by UV curing to transform the microdroplets into hydrogel microspheres [49]. The curing time of microspheres in this study, which is primarily determined by material properties, requires evaluation to ensure continuous production using the microfluidic chip. Furthermore, the microspheres prepared in this study have a size range of approximately 100 to 300 μm.

The environmental responsiveness of conjugates refers to the ability of the materials to exhibit changes or adjustments in their physical or chemical properties in response to external environmental variations [50]. In the case of rapidly progressing OA, there is a gradual increase in the acidity of the joint fluid. The Schiff base bonds, which anchor CST-14 to the microsphere surface, undergo decomposition in an acidic environment [47]. As a result, the drug is rapidly released from the microsphere surface after losing its anchoring, effectively suppressing the progression of the disease. In the case of a slow progression of the disease, the stable anchoring of CST-14 onto the hydrogel network of microspheres prevents its complete release in the early stages of disease progression, thereby minimizing potential loss or wastage.

We successfully used Schiff base bonds to construct a biomimetic conjugate in a simple and controllable manner, which holds promise for real-time dynamic therapy and the prevention of OA progression. The various monomeric components of this biomimetic conjugate play multifaceted and crucial roles. The hydrogel developed in this study takes the form of microspheres, which means that it has a larger specific surface area compared to the same volume, allowing it to adsorb more medication and be easier to inject. It provides lubrication for articular cartilage in the form of spherical gels. The gel microspheres comprise a double network; the PEGDA gel network solidifies the droplets into microspheres, acting as a skeleton. DFPEG and chitosan form the second gel network, enhancing the mechanical properties of the gel microspheres and providing them with high environmental sensitivity. The second network enriches the gel network surface with many active aldehyde groups, which can fix CTS-14 for the treatment of arthritis and achieve dynamic release according to the progression of inflammation. The conjugate not only provides a novel approach for anchoring amino-containing drugs but also enables the reuse of the drug delivery system. In the context of severe OA, targeted drug therapy for inflamed tissues and self-regulation of the gel network have been achieved. When OA occurs, the localized acidification of the damaged cartilage surface causes rapid destruction of the drug-anchoring structure, similar to a “strong wind”. This is further suppressed by dispersing the “seeds” of CST-14, which inhibits the progression of the disease, while the re-exposed active aldehyde groups protect the tissue by inhibiting oxidation. Once the disease is suppressed, the local acidic environment is relieved, allowing the high-energy covalent bonds to be more stable than amide or disulfide bonds, which allows for prolonged drug delivery time, waiting for the next “strong wind” to arrive. Important therapeutic effects were observed in both in vitro and in vivo experiments. The results demonstrated that the biomimetic conjugates exhibit excellent biocompatibility, acid-responsive characteristics, lubricating properties, and the ability to suppress inflammatory responses in chondrocytes while providing cellular protection by antagonizing oxidative stress. This study offers a novel approach for further therapeutic interventions in OA.

## Data Availability

The datasets used and/or analyzed during the current study are available from the corresponding author on reasonable request.
