# Supplementary material for: Nanoarchitectonics of Injectable Biomimetic Conjugates for Cartilage Protection and Therapy Based on Degenerative Osteoarthritis Progression
Source: Biomater Res. 2024 Sep 10;28:0075. doi: 10.34133/bmr.0075 (PMC11383433; doi:10.34133/bmr.0075)
Supplement: Supplementary 1 — Fig. S1 Tables S1 to S6 [file bmr.0075.f1.zip › Supplementary Material.docx]

**Injectable biomimetic conjugates achieve targeted therapy and cartilage protection based on degenerative osteoarthritis progression**

*Jingwei Bi ^a,§^, Limin Zhang ^b,§^, Pengfei Zhang ^a,§^, Shulei Xu ^b^, Yuhao Liu ^a^, Xiaolai Zhang ^c^, Xiaoyong Qiu ^c^, Yanwen Bi ^d^,Fangfang Yan ^e^, Hui Wei ^f^, Xin Cui ^g^, Xin Pan ^a ,*^, Jun Huang ^b,*^, Yunpeng Zhao ^a,*^*

*^a^ Department of Orthopaedic, Qilu Hospital of Shandong University, Jinan, Shandong, 250012, China;*

*^b^ Center for Advanced Jet Engineering Technologies (CaJET), Key Laboratory of High Efficiency and Clean Mechanical Manufacture of Ministry of Education, School of Mechanical Engineering, Shandong University, Jinan, Shandong, 25006, China*

*^c^ Key Laboratory of Colloid and Interface Chemistry of the Ministry of Education,* *School of Chemistry and Chemical Engineering, Shandong University, Jinan 250100, China*

*^d^ Department of Cardiovascular Surgery, Qilu Hospital of Shandong University,*

*Jinan, Shandong 250012, China*

*^e^ Department of Traditional Chinese Medicine, Qilu Hospital of Shandong University,*

*Jinan, Shandong 250012, China*

*^f^ Rehabilitation Center, Qilu Hospital of Shandong University, Jinan, Shandong 250012, China*

*^g^ Advanced Interdisciplinary Technology Research Center, National Innovation Institute of Defense Technology, Beijing, 100071, China*

*^§^These authors contributed equally to this work.*

**Corresponding authors. panxin0714@sina.com (X. P.); jun.huang@email.sdu.edu.cn (J. H.); lwwzyp@email.sdu.edu.cn (Y. Z.)*





Fig. S1 Maximum compression strain of the PEGDA&C&DFPEG hydrogel after being immersed in deionized water for 5 min and 12 h. The compression tests were repeated three times to check the reproducibility.

**Table S1**. Parameters of the solution condition used in this study

| Parameters |  |
| --- | --- |
| The flow rate of aqueous phase (ml /min) | 0.005 |
| The flow rate of oil phase (ml /min) | 0.05, 0.10, 0.15, 0.20, 0.25 |
| Contact angle (°) | 90 |
| The viscosity of aqueous solution ($Pa\cdot s$) | 0.02954 |
| The density of aqueous solution (kg/m³) | 1004 |
| The viscosity of oil solution ($Pa\cdot s$) | 0.0013 |
| The density of oil solution (kg/m³) | 644 |
| Interfacial tension (N/m) | 0.0021254 |

**Table S2.** Composition list of different samples used for NMR spectroscopy analysis

| Sample number | Cortistatin-14  (mg) | DFPEG  (mg) | Preparation process | Storage temperature |
| --- | --- | --- | --- | --- |
| 1 | 1 | 3 | Samples freeze-dried post-reaction. | -64℃ |
| 2 | 0 | 3 | - | -64℃ |
| 2 | 1 | 0 | - | -20℃ |

**Table S3.** Composition list of different samples used for rheological performance testing.

| Sample number | PEGDA content  (wt %) | Chitosan content  (wt %) | Irgacure 2959 content (wt %) | Glacial acetic acid content  (wt %) | DFPEG  content  (wt %) |
| --- | --- | --- | --- | --- | --- |
| 1 | 10 | 0 | 2. | 0 | 0 |
| 2 | 10 | 0.5 | 2 | 0.5 | 0 |
| 3 | 11 | 0.5 | 2 | 0.5 | 1 |

**Table S4.** Absorbance values of different groups on the first day.

| BLANK (OD) | CTL (OD) | Microspheres (OD) | CST@Microspheres (OD) |
| --- | --- | --- | --- |
| 0.043 | 0.464 | 0.553 | 0.563 |
| 0.044 | 0.536 | 0.533 | 0.513 |
| 0.043 | 0.494 | 0.645 | 0.595 |

**Table S5.** Absorbance values of different groups on the second day.

| BLANK (OD) | CTL (OD) | Microspheres (OD) | CST@Microspheres (OD) |
| --- | --- | --- | --- |
| 0.041 | 0.504 | 0.597 | 0.607 |
| 0.038 | 0.579 | 0.59 | 0.606 |
| 0.043 | 0.504 | 0.544 | 0.592 |

**Table S6.** Absorbance values of different groups on the third day.

| BLANK (OD) | CTL (OD) | Microspheres (OD) | CST@Microspheres (OD) |
| --- | --- | --- | --- |
| 0.05 | 1.112 | 1.353 | 1.336 |
| 0.05 | 1.037 | 1.337 | 1.286 |
| 0.047 | 1.076 | 1.312 | 1.471 |
